# Peroxidasin Enhances Basal Phenotype and Inhibits Branching Morphogenesis in Breast Epithelial Progenitor Cell Line D492

**DOI:** 10.1007/s10911-021-09507-1

**Published:** 2021-12-28

**Authors:** Anna Karen Sigurdardottir, Arna Steinunn Jonasdottir, Arni Asbjarnarson, Hildur Run Helgudottir, Thorarinn Gudjonsson, Gunnhildur Asta Traustadottir

**Affiliations:** 1 Stem Cell Research Unit, Biomedical Center, Department of Anatomy, Faculty of Medicine, School of Health Sciences, University of Iceland, Reykjavik, Iceland; 2 Department of Laboratory Haematology, Landspitali – University Hospital, Reykjavik, Iceland

**Keywords:** Peroxidasin, p63, Mammary gland, Branching morphogenesis, D492, Mammary stem cells

## Abstract

The human breast is composed of terminal duct lobular units (TDLUs) that are surrounded by stroma. In the TDLUs, basement membrane separates the stroma from the epithelial compartment, which is divided into an inner layer of luminal epithelial cells and an outer layer of myoepithelial cells. Stem cells and progenitor cells also reside within the epithelium and drive a continuous cycle of gland remodelling that occurs throughout the reproductive period. D492 is an epithelial cell line originally isolated from the stem cell population of the breast and generates both luminal and myoepithelial cells in culture. When D492 cells are embedded into 3D reconstituted basement membrane matrix (3D-rBM) they form branching colonies mimicking the TDLUs of the breast, thereby providing a well-suited *in vitro* model for studies on branching morphogenesis and breast development. Peroxidasin (*PXDN*) is a heme-containing peroxidase that crosslinks collagen IV with the formation of sulfilimine bonds. Previous studies indicate that *PXDN* plays an integral role in basement membrane stabilisation by crosslinking collagen IV and as such contributes to epithelial integrity. Although *PXDN* has been linked to fibrosis and cancer in some organs there is limited information on its role in development, including in the breast. In this study, we demonstrate expression of PXDN in breast epithelium and stroma and apply the D492 cell line to investigate the role of *PXDN* in cell differentiation and branching morphogenesis in the human breast. Overexpression of *PXDN* induced basal phenotype in D492 cells, loss of plasticity and inhibition of epithelial-to-mesenchymal transition as is displayed by complete inhibition of branching morphogenesis in 3D culture. This is supported by results from RNA-sequencing which show significant enrichment in genes involved in epithelial differentiation along with significant negative enrichment of EMT factors. Taken together, we provide evidence for a novel role of *PXDN* in breast epithelial differentiation and mammary gland development.

**Supplementary Information:**

The online version contains supplementary material available at 10.1007/s10911-021-09507-1.

## Introduction

The mammary gland, the signature organ of mammals, is responsible for the production of milk to feed the offspring. The gland is a branched structure comprised of excretory ducts and terminal duct lobular units (TDLUs) which contain the functional epithelium along with stroma. The epithelium can be divided into an inner layer of luminal epithelial cells (LEP) and an outer layer of myoepithelial cells (MEP), both believed to be of the same origin [1–3]. Separating the epithelium from the stroma is the basement membrane, a thin layer of extracellular matrix mainly comprised of laminins and collagen IV, that maintains epithelial integrity and induces correct cell polarity [4, 5]. Although the development of the mammary gland begins during embryogenesis it is unique in that it does not complete its developmental process until pregnancy. However, during each oestrus cycle, continuous epithelial remodelling occurs as the gland prepares in prospect of pregnancy [6, 7]. This remodelling is maintained by bipotent epithelial stem cells that, along with lineage restricted progenitor cells, induce branching and epithelial differentiation within the gland [2, 8–10]. Branching morphogenesis is dependent upon epithelial cell plasticity that enables the cells to invade the surrounding stroma. This can be achieved through mechanisms such as collective migration [11] or epithelial to mesenchymal transition (EMT) [12, 13].

The D492 breast epithelial progenitor cell line was originally isolated from the EpCAM^+^/MUC1^-^ supra-basal cell population of the human breast epithelium [1]. This population is multipotent and can give rise to cells with both luminal and myoepithelial characteristics when cultured *in vitro*. This ability is preserved in D492 cells which, furthermore, mimic the breast TDLUs by forming branched structures when cultured in 3D reconstituted basement membrane (3D-rBM, Matrigel) [1]. In addition, D492 cells undergo EMT when co-cultured with endothelial cells [14]. Due to its ability to replicate key features of breast development the D492 cells are well suited for *in vitro* modelling and have successfully been used to study branching morphogenesis, epithelial plasticity and EMT [14, 15]. Furthermore, D492 has been used to study the role of non-coding RNAs (nc-RNAs) in these processes [16–19].

Recently, we applied D492 and its mesenchymal daughter cell line D492M to study differential expression of miRNAs during EMT. This revealed that miR-203a was one of the most downregulated miRNAs in D492M compared to D492. Subsequent overexpression of miR-203a in D492M led to partial revision to an epithelial phenotype with accompanying functional changes such as decreased proliferation, migration and invasion, and increased susceptibility to chemically induced apoptosis. Interestingly, RNA-sequencing revealed that the most downregulated gene in D492M^miR-203a^ was the basement membrane factor peroxidasin (*PXDN*) which we subsequently confirmed as a novel target for miR-203a [17]. Peroxidasin (*PXDN*) is an extracellular matrix peroxidase and the only known enzyme that stabilizes the basement membrane through sulfilimine bond formation between collagen IV fibrils [20] which are only found within the basement membrane [21]. *PXDN* loss of function leads to basement membrane destabilisation due to lack of cross-linking, indicating that *PXDN* plays a crucial role in development and tissue homeostasis [20, 22]. *PXDN* is expressed in various tissues such as the heart, colon and liver, and on the cellular level it has been identified in epithelium, vascular endothelium and smooth muscle cells [23]. In melanoma, increased *PXDN* expression has been linked to mesenchymal and invasive phenotype of malignant cells [24]. Furthermore, high *PXDN* expression in ovarian cancer has been linked to increased malignancy and poor patient outcome. Conversely, *in vitro* knock down of *PXDN* in HEY human ovarian carcinoma cell line decreased invasion, migration and proliferation, supporting the link between *PXDN* and malignant phenotype [25]. In the present study, we addressed the largely unexplored functional role of *PXDN* in mammary gland development by overexpressing the *PXDN* gene in the normal epithelial progenitor cell line D492. We demonstrate that overexpression of *PXDN* in D492 cells enforces basal epithelial phenotype and inhibits branching. Furthermore, we show through RNA-sequencing that *PXDN* is involved in epithelial cell differentiation and inhibition of EMT.

## Materials and Methods

### D492 Cell Culture

D492 were cultured in H14 media containing Dulbecco‘s Modified Eagle Medium DMEM:F12 (Gibco, #31330), supplemented with penicillin and streptomycin, Insulin (Sigma, #I1882), EGF (Peprotech, #AF-10-15), Transferrin (Sigma, #T1147), NaSel (BD Biosciences, #354201), Estradiol (Sigma, #E2758), Hydrocortisone (Sigma, #H0888), and Prolactin (Peprotech, #100-07). Cells were cultured in flasks precoated with collagen I solution (PureCol Type 1 Collagen Solution, Advanced Biomatrix, #5005-100ML) at 37 °C and 5% CO_2_. Media was changed three times a week and cells were passaged when 90% confluent at a ratio of 1:10.

### 3D Matrix Embedded Culture

3D cultures with cells embedded in matrix were carried out in 48 well plates (Corning, #353078), where 1,0x10^4^ cells were resuspended and plated in 150 µl of growth factor reduced reconstituted basement membrane matrix (Matrigel, Corning, #354230) in triplicates. Plate was incubated at 37 °C and in 5% CO_2_ for 30 minutes until Matrigel had solidified, and 500 µl of H14 media was gently added on top. Cells were grown for 21 days, and media was changed three times a week.

### 3D on Top of Matrix Culture

3D with cells on top of matrix was carried out in 96 well plates (Matrigel, Corning, #354230), where 50 µl of Matrigel were plated and then incubated for 30 minutes at 37 °C and in 5% CO_2_ until the gel had solidified. Subsequently, 6,0x10^3^ cells were seeded on top of the gel in 100 µl of H14 media. Media was changed three times a week by replacing half of old media with equal volume of fresh media. Cells were grown for 8 days, and colony formation and growth were monitored in IncuCyte Live Cell Analysis System (Essen BioScience).

### Isolation of Primary Cells from Breast Tissue

Luminal epithelial- and myoepithelial cells were purified after breast organoids from reduction mammoplasty had spread out in primary culture. The isolation was done immunomagnettically using anti-EpCAM columns. Luminal- and myoepithelial cells separations were carried out by use of the MiniMACS magnetic cell separation system according to the manufacturer’s instructions (Miltenyi Biotech) [1]. BRENCs were isolated from adipose tissue derived from reduction mammoplasty which resulted in relatively pure microvessel organoids. The microvasculature component was incubated with anti-CD31 Dynabeads (Invitrogen, #11155D) and isolated on a magnetic concentrator. Microvessels were seeded onto collagen coated flasks, resulting in enriched population of endothelial cells [26]. Fibroblasts appear as single cells upon collagenase treatment of the breast tissue. Purification of fibroblasts was obtained by differential centrifugation of the digest and moving the floating fibroblast to a culture flask [27].

### Lentiviral Packaging and Transduction

Lentiviral particles were produced with pLenti-C-Myc-DDK-P2A-Puro cloning vector containing *PXDN* sequence (OriGene, #RC224518L3) along with pMD2.G (a gift from Didier Trono, Addgene plasmid #12259; http://n2t.net/addgene:12259; PRID:Addgene_12259) and psPAX2 (a gift from Didier Trono, Addgene plasmid #12260; http://n2t.net/addgene:12260; PRID:Addgene_12260) packaging plasmids. Plasmids were transfected into HEK-293T cells using TurboFect Transfection Reagent (Thermo Fisher Scientific, #R0533) in antibiotic and serum free high glucose DMEM (Gibco, #31966-021). Supernatant containing lentivirus was collected 48 hours and 72 hours post-transfection. D492 were infected with viral supernatant overnight in the presence of 8 µg/ml Polybrene (Sigma-Aldrich, #TR-1003). *PXDN* transduced cells were subsequently selected with puromycin (Gibco, #A11138-03) at concentration of 2 µg/ml for D492 cells. Control cells were transduced with empty pLenti-C-Myc-DDK-P2A-Puro cloning vector (OriGene, #PS100092).

### RNA Isolation and Quantitative RT-PCR Analysis

RNA was isolated with Tri-Reagent (Thermo Fisher Scientific, #AM9738) and 1 µg of each sample RNA was reverse transcribed using LunaScript RT SuperMix Kit (New England BioLabs, #E3010). Quantitative real time PCR was performed using SYBR Green Luna Universal qPCR Master Mix (New England BioLabs, #M3003) as per manufacturer recommendation. Relative expression was determined via calculation of 2^-ΔΔCt^ using ABI 7500 instrument (Applied Biosystems) and GAPDH as endogenous control for gene expression. The following primers were used (purchased from IDT Integrated DNA Technologies) *PXDN* (Hs.PT.58.630748)*, CK14* (Hs.PT.58.4592110)*, CK19* (Hs.PT.58.4188708)*, CK5/6* (Hs.Pt.58.14446018)*, TP63* (Hs.PT.58.2966111) and *GAPDH* (Hs.Pt.39a.22214836) as an endogenous control.

### Immunochemistry of Cells

For immunochemistry of cells in monolayer and 3D culture the following antibodies were used: PXDN (a gift from M. Geiszt, Semmelweis University, Budapest) 1:250, KRT19 (Abcam, #ab7754) 1:100, KRT14 (Abcam, #ab7800) 1:100, and p63 (Novocastra, #NCL-p63) 1:100. Fluorescent labelling was performed using fluorescent secondary antibodies (Alexa fluor, Thermo Fisher Scientific). Imaging was performed on Zeiss LSM 5 Pa laser-scanning microscope (Carl Zeiss) and Olympus fluoview 1200.

### Immunohistochemistry of Paraffin Embedded Mammary and Breast Cancer Tissue

Immunohistochemistry of tissue slides was performed with anti-PXDN (a gift from M. Geiszt, Semmelweis University, Budapest) 1:400. Secondary staining was performed with Fast Red (Sigma, #F4648). Tissue slides were counterstained with hematoxylin. Imaging was performed using Nikon Eclipse Ci microscope.

### Protein Isolation and Western Blot Assay

Protein was isolated from cells lysed with radio immunoprecipitation assay (RIPA) buffer containing protease and phosphatase inhibitors (Halt Protease Inhibitor Cocktail, Thermo Fisher Scientific, #78430). Bradford reagent (BioRad, #5000002) was used to determine protein concentration. Western blot was performed on 10% NuPage Bis-Tris gels (Invitrogen, #NP0301PK2) in NuPage MES running buffer (Thermo Fisher Scientific, #NP0002). Protein was transferred from gel to Immobilon-FL PVDF membrane (Merck-Millipore, #IPFL00010) with NuPage transfer buffer (Thermo Fisher Scientific, #NP0006-1). Membrane was blocked with Odyssey Blocking Buffer (Li-Cor, #927-500) and incubated overnight at 4 °C with primary antibodies. The following primary antibodies were used for protein detection: PXDN (a gift from M. Geiszt, Semmelweis University, Budapest) 1:1000, KRT19 (Santacruz, #sc-6278) 1:1000, KRT14 (Abcam, #ab7800) 1:1000, KRT5/6 (Invitrogen, #180267) 1:500, EpCam (Abcam, #ab71916) 1:1000, with Actin (Li-Cor, #924-42212) as endogenous control, and in nuclear fraction p63 (Abcam, #ab124762) 1:2000, with Histone H3 (Cell Signaling, #4499). Secondary antibodies used were rabbit or mouse (Li-Cor) and imaging was performed using Odyssey Infrared Imaging System (Li-Cor).

### Proliferation Assay

Cells were seeded at 4.0x10^3^/cm^2^ in a 6-well plate (Falcon, #353046) format, pre-coated with collagen I solution (PureCol Type 1 Collagen Solution, Advanced Biomatrix, #5005-100ML). Plates were incubated at 37 °C and 5% CO_2_. Every 24 hours three wells of each cell line were treated with Trypsin-EDTA, and cells were counted. Proliferation rate was determined from increase in cell number over six consecutive days.

### Apoptosis Assay

Apoptosis assay was performed in IncuCyte (Essen Bioscience) as per manufacturer’s instructions using 10µM Camptotechin (Sigma-Aldrich, #C9911). Apoptosis was detected by IncuCyte Caspase-3/7 Green Reagent (Essen Bioscience, #4440).

### Migration and Invasion Assays

Migration and invasion assays were performed as per manufacturer’s instructions in IncuCyte (Essen Bioscience) in a 96 well plate format. 1.0x10^10^ cells were seeded per well and allowed to grow until confluent before scratching.

### Statistical Analysis

Statistical analysis was performed in GraphPad Prism version 8.3.0. All experiments were performed in three independent experiments.

### RNA Sequencing

RNA was isolated from D492^empty^ and D492^*PXDN*^ cells in monolayer at 80% confluency and on day 8 of 3D on top of matrix culture. RNA sequencing was performed on samples in quintuplets at DeCODE Genetics (Reykjavik, Iceland).

Gene enrichment analyses were performed in GSEA (gsea-msigdb.org) with pre-ranked gene lists and the Hallmark and C5 Ontology gene sets for HALLMARK and GO analyses, respectively. Volcano plots and heatmaps were generated in Graph Pad Prism.

### Transient Knock Down of *TP63 *and *PXDN*

Transient knock down in D492^PXDN^ cells was performed with siRNA targeting *TP63* (Thermo Fisher Scientific. SiRNA #1: #s16411 lot.AS02HN1T. SiRNA #2: #s502043 lot.AS02043), and in D492 cells with siRNA targeting *PXDN* (Thermo Fisher Scientific, #4392421 lot.AS02B0C4), using Lipofectamine RNAiMAX Transfection Reagent (Thermo Fisher Scientific, #13778075) as per manufacturer’s instructions. Cells were incubated for 48 hours after transfection whereafter assays were performed.

### Analysis of *PXDN* Expression in Breast Cancer

*PXDN* expression in different breast cancer subtypes was analysed in the Gene expression-based Outcome for Breast Cancer Online (GOBO) database [28]. Distant metastasis free survival (DMFS) over a period of 10 years was analysed in 3 quantiles in all tumor subtypes.

## Results

### *PXDN* is Expressed in Breast Epithelium and Stroma

**Fig. 1:**
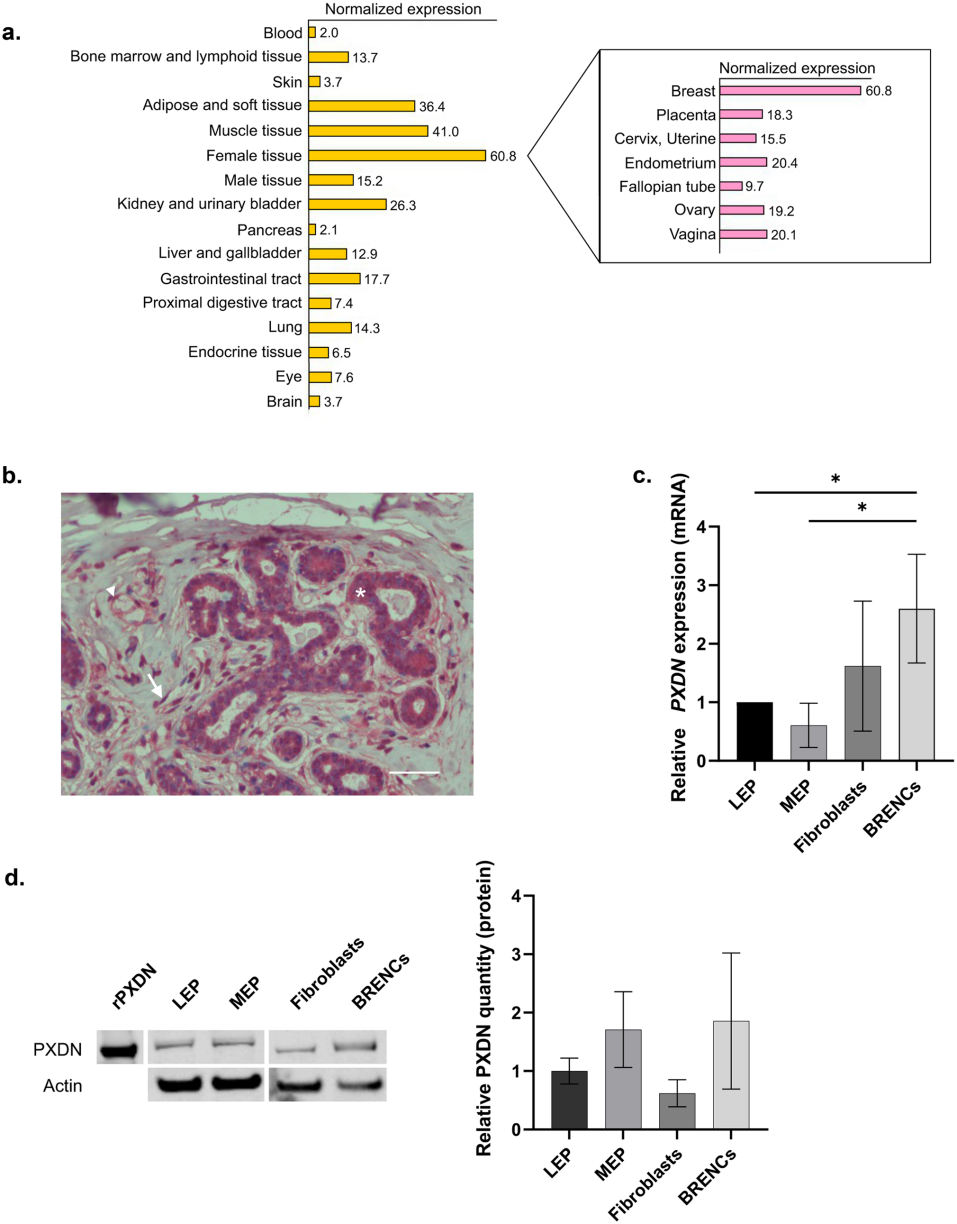
*PXDN* is expressed in both epithelial and stromal compartments of the breast gland. **a** *PXDN* expression is highest in breast tissue. Tissue specific expression data from the Human Protein Atlas showed that *PXDN* levels are higher in breast than other tissues. **b** Immunohistochemistry of PXDN in paraffin embedded normal breast tissue. Positive signal (red) was detected in epithelial cells (example depicted with asterix), fibroblasts (example depicted with arrow) and endothelial cells (example depicted with arrowhead). Tissue slides were counterstained with hematoxylin. Scale bar = 100 µm. **c** *PXDN* was expressed in luminal epithelial cells (LEP), myoepithelial cells (MEP), fibroblasts and breast endothelial cells (BRENCs), isolated from primary tissue from reduction mammoplasties. BRENCs expressed the highest levels of *PXDN* compared to the other three subtypes followed by fibroblasts, as detected by qRT-PCR. Furthermore, *PXDN* was expressed in both LEPs and MEPs which both belong to the epithelial compartment. Statistical significance was determined by One-way ANOVA (*p ≤ 0.05) and data is presented as an average of three replicated experiments (mean ± SD). **d** Western blot showing protein level expression of PXDN in LEP, MEP, fibroblasts and BRENCs with recombinant PXDN (rPXDN) as positive control. No significant difference was found in PXDN expression between primary cells. Statistical significance was determined by One-way ANOVA and data is presented as an average of three replicated experiments (mean ± SD)

Data from the Human Protein Atlas database revealed that *PXDN* expression is highest in female tissues compared to other human tissues and among those, *PXDN* expression was highest in the breast (Fig. 1a). In support, immunohistochemistry of paraffin embedded normal breast tissue revealed positive PXDN signal in epithelial cells, fibroblasts and endothelial cells (Fig. 1b). To confirm the expression of *PXDN* in the mammary gland we investigated the expression pattern in four different subtypes of primary cells isolated from reduction mammoplasties: EpCam-sorted luminal epithelial cells (LEPs) and myoepithelial cells (MEPs) (Supplementary Fig. 1), fibroblasts and breast endothelial cells (BRENCs). Quantitative RT-PCR analysis revealed that *PXDN* was expressed in all cell types, albeit with higher expression in fibroblasts and BRENCs, both belonging to the breast stromal compartment. Considerable expression was also detected in LEPs and MEPs, indicating a role of *PXDN* within the functional epithelium. (Fig. 1c). Western blot confirmed protein level expression in all four primary cell types, although the results did not completely reflect mRNA-level expression (Fig. 1d).

### Overexpression of *PXDN* in D492 Affected Monolayer Growth Pattern, Enforced Basal Epithelial Phenotype and Inhibited Branching Morphogenesis

**Fig. 2:**
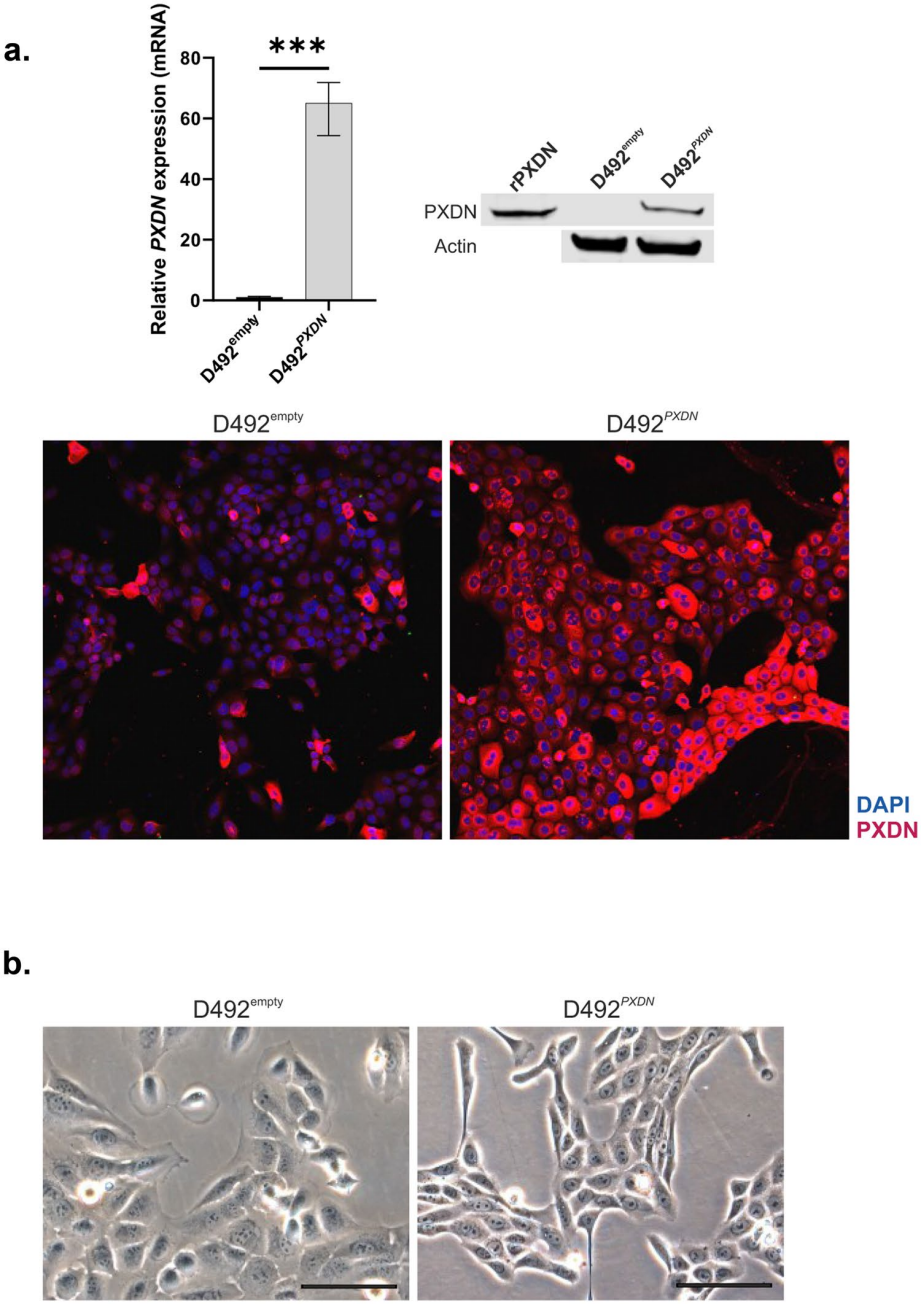
Confirmation of *PXDN* overexpression in D492 cells and effect on monolayer growth pattern. **a** *PXDN* was successfully overexpressed in D492. qRT-PCR results showed approximately 65-fold upregulation of *PXDN* in D492^*PXDN*^ compared to D492^empty^. Results were confirmed by Western blot where recombinant *PXDN* (rPXDN) was used as positive control, and by immunofluorescence staining. Statistical significance in qRT-PCR was determined by unpaired Student‘s t-test (***p ≤ 0.001) and data is presented as an average of three replicated experiments (mean ± SD). Scale bar = 125 µm. **b** Overexpression of *PXDN* affected cell phenotype and growth pattern of D492 in monolayer culture. D492^*PXDN*^ cells were more homogeneous and cuboidal in phenotype compared to D492^empty^. D492^*PXDN*^ grew closer together than D492^empty^ in monolayer, forming a dense network as opposed to more even distribution of cells in D492^empty^. Scale bar = 100 µm

We have previously reported that while *PXDN* is minimally expressed in D492 it is significantly upregulated in its mesenchymal daughter cell line D492M [17]. As D492 cells are well established as a model for branching morphogenesis in the breast and yield cells with both luminal and myoepithelial characteristics, we decided to apply D492 to investigate the role of *PXDN* in mammary gland development and epithelial differentiation. Therefore, we overexpressed *PXDN* in D492 cells using lentiviral approach. Quantitative RT-PCR revealed approximately 65-fold upregulation of *PXDN* in D492^*PXDN*^ compared to D492^empty^ and successful upregulation was confirmed with Western blot and immunofluorescence staining of monolayer cell culture (Fig. 2a). In monolayer culture, D492^*PXDN*^ cells were phenotypically distinguishable from D492^empty^ cells, as they were smaller, cuboidal and more homogeneous in appearance compared to D492^empty^. Furthermore, they grew in tighter proximity to neighbouring cells than D492^empty^ (Fig. 2b).

**Fig. 3:**
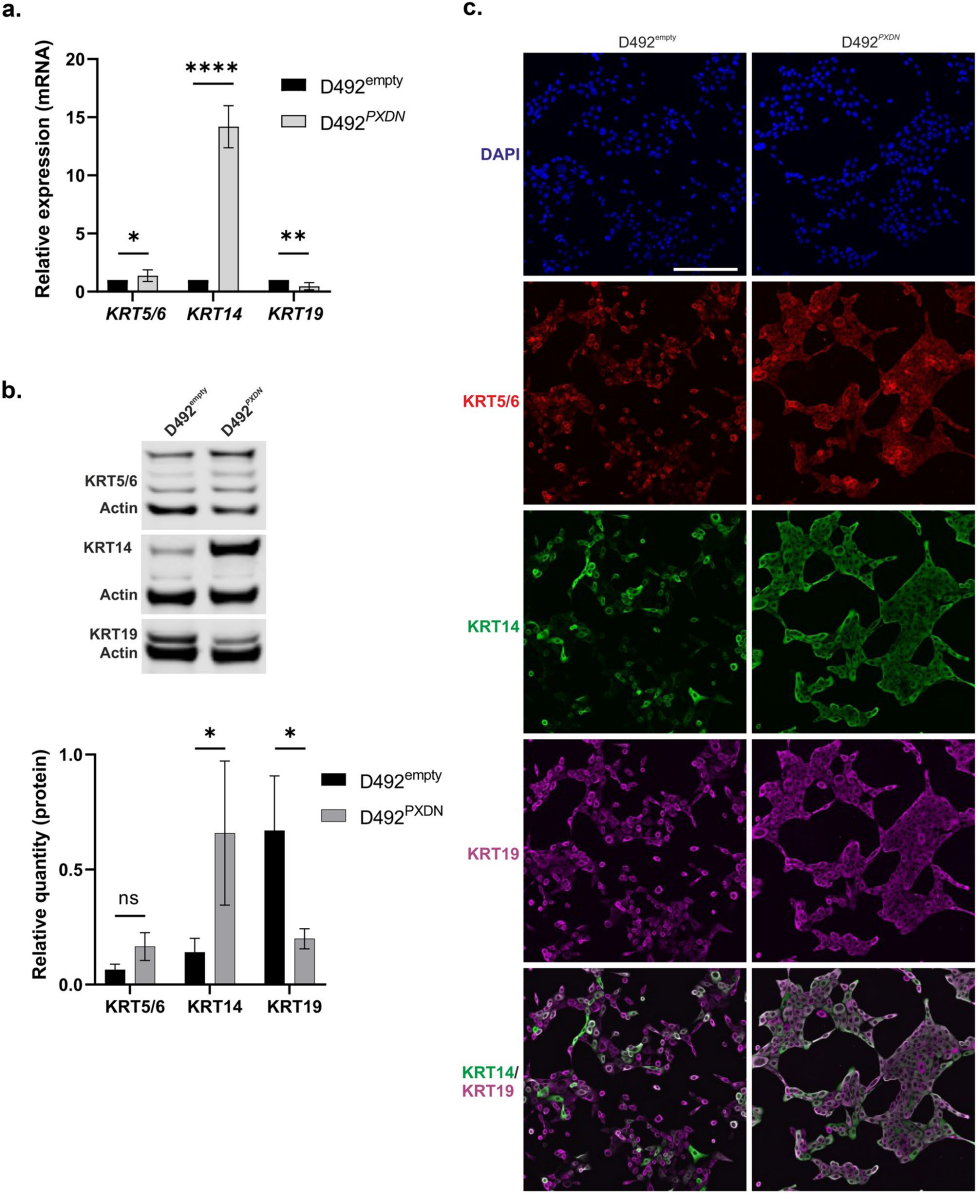
Overexpression of *PXDN* in D492 enforced epithelial basal phenotype. **a** Overexpression of *PXDN* enforced upregulation of basal epithelial cell markers in D492. qRT-PCR results revealed that epithelial basal cell markers *KRT14* and *KRT5/6* were significantly upregulated in D492^*PXDN*^ compared to D492^empty^, while luminal epithelial cell marker *KRT19* was significantly downregulated. Statistical significance in qRT-PCR was determined by unpaired Student‘s t-test (*p ≤ 0.05, **p ≤ 0.01, ****p ≤ 0.0001) and data is presented as an average of three replicated experiments (mean ± SD). Scale bar = 125 µm. **b** Western blot confirmed protein level upregulation of basal markers in D492^*PXDN*^. Relative quantification of protein bands confirmed significant upregulation of *KRT5/6*, *KRT14* and *KRT19*. Statistical significance was determined by unpaired Student‘s t-test (*p ≤ 0.05) and data is presented as an average of three replicated experiments (mean ± SD). **c** D492^*PXDN*^ cells coexpressed luminal and basal markers. Immunofluorescence staining revealed that D492^empty^ had both *KRT14-/KRT19+* and *KRT14+/KRT19+* populations. In D492^*PXDN*^ the *KRT14-/KRT19+* population was lost while *KRT14+/KRT19+* cells were enriched. Scale bar = 125 µm

Having established phenotypic differences between D492^*PXDN*^ and D492^empty^, we asked whether this was reflected by changes in epithelial cell differentiation markers. Indeed, quantitative RT-PCR showed significant upregulation of basal markers *KRT14* and *KRT5/6* in D492^*PXDN*^, while luminal marker *KRT19* was significantly downregulated (Fig. 3a). Differential expression of these three markers was confirmed on protein level by Western blotting where significant change in expression of all three markers was observed (Fig. 3b). Immunofluorescence staining of cells cultured in monolayer revealed upregulation of both KRT5/6 and KRT14 in D492^*PXDN*^ while changes in expression of KRT19 were less clear. However, when comparing the expression pattern of KRT14 and KRT19 it was apparent that all D492^*PXDN*^ cells co-expressed both markers and that there was a depletion of KRT19+/KRT14- cells (Fig. 3c). This subpopulation of KRT19+/KRT14*-* cells reflected the luminal population of the breast epithelium [29] while it has been suggested that KRT19+/KRT14+ cells are mammary gland stem cells [2]. The ability to yield populations that are KRT19+/KRT14*-* or KRT19-/KRT14+ with luminal and myoepithelial characteristics, respectively, in addition to a small KRT19+/KRT14+ subpopulation is characteristic for the D492 cell line and is reflective of its plasticity [15]. The depletion of populations other than KRT19+/KRT14*+* in D492^*PXDN*^ might therefore indicate loss of plasticity. To analyse whether overexpression affected the expression of *COL4A1* and *COL4A5* we decided to evaluate the expression in D492^empty^ and D492^*PXDN*^ which revealed no observed differences between the cell lines (Supplementary Fig. 2).

**Fig. 4:**
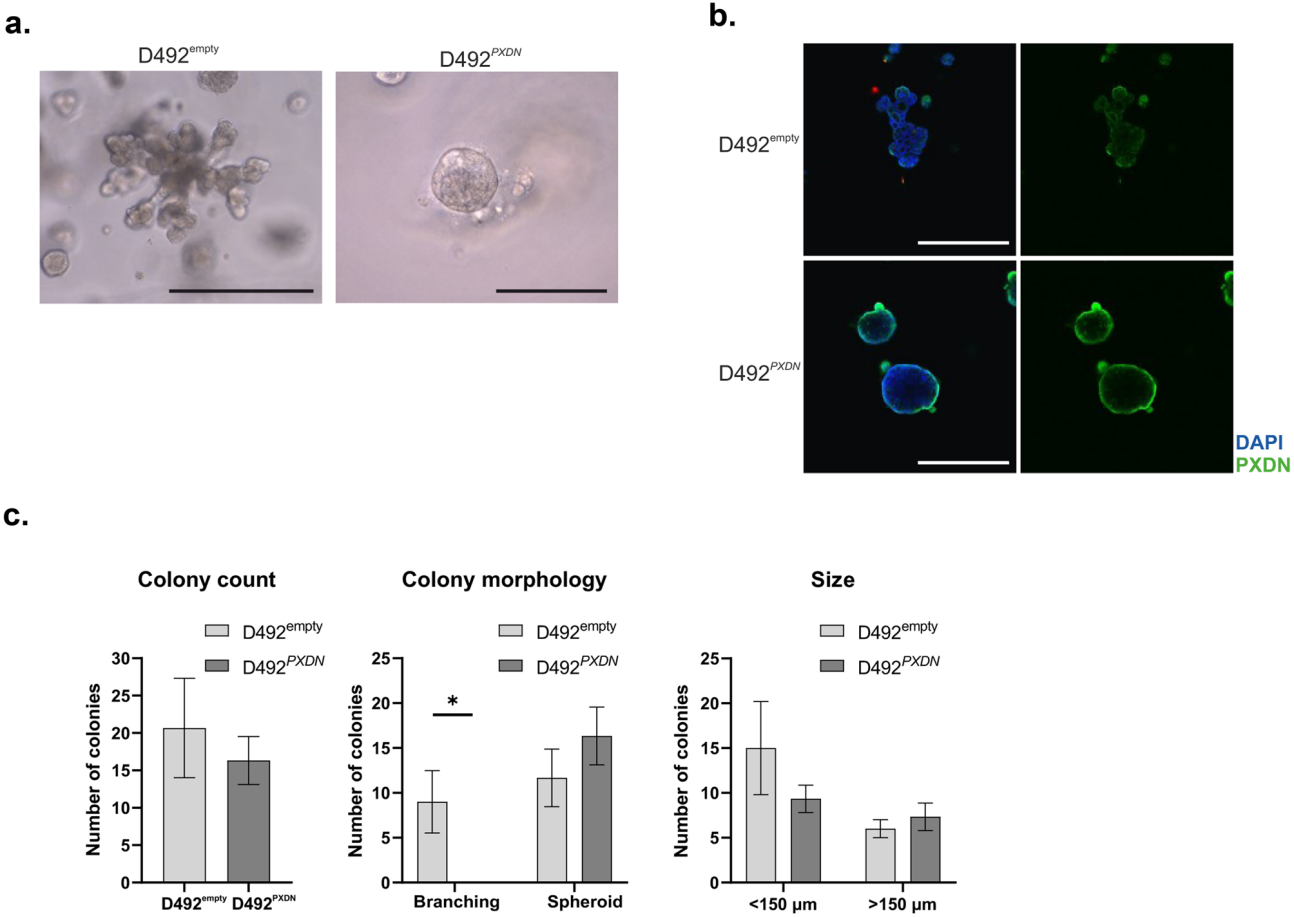
*PXDN* inhibited branching morphogenesis in D492 cells. **a** When cultured in 3D-rBM for 21 days, D492 cells form branching structures reminiscent of TDLUs in the breast. This ability was retained in D492^empty^. However, branching morphogenesis was completely inhibited in D492^*PXDN*^ cells. Scale bar = 125 µm. **b** Overexpression of *PXDN* in D492^*PXDN*^ was preserved under 3D conditions. Increased secretion of *PXDN* to the ECM was observed in D492^*PXDN*^ cells cultured in 3D-rBM. *PXDN* was also visible around D492^empty^ colonies but to a lesser extent. Scale bar = 125 µm. **c** Overexpression of *PXDN* significantly altered morphology but not count and size of colonies in 3D-on top culture. No significant difference was seen in colony count or size between to D492^empty^ and D492^*PXDN*^ cultured on top of Matrigel on day 9. However, branching was inhibited in D492^*PXDN*^, which only formed solid spheroid colonies. Scale bar = 150 µm. Statistical significance was determined using unpaired Student‘s t-test and data is presented as an average of three replicated experiments (mean ± SD)

We have previously demonstrated that plasticity is necessary for branching morphogenesis to occur in D492 cultured in 3D-rBM [16]. Colonies formed by cells with either luminal or myoepithelial phenotype without plasticity were unable to adjust their phenotype and thus, were also unable to branch [16]. Interestingly, 3D cultures of D492^empty^ and D492^*PXDN*^ showed that D492^empty^ colonies retained the branching ability of D492, whereas branching was completely inhibited in D492^*PXDN*^ (Fig. 4a). Immunohistochemical staining of D492^*PXDN*^ and D492^empty^ 3D colonies revealed that increased expression of *PXDN* was preserved under 3D conditions (Fig. 4b). Colony characterisation confirmed that there was no significant difference between the size and number of D492^empty^ and D492^*PXDN*^. Colony morphology reflected previous observation where D492^empty^ formed both spheroid and branching colonies while D492^*PXDN*^ only formed spheroids (Fig. 4c).

### Overexpression of *PXDN* Decreased Wound Closure Times in D492 Cells and Negatively Affected Plasticity

**Fig. 5:**
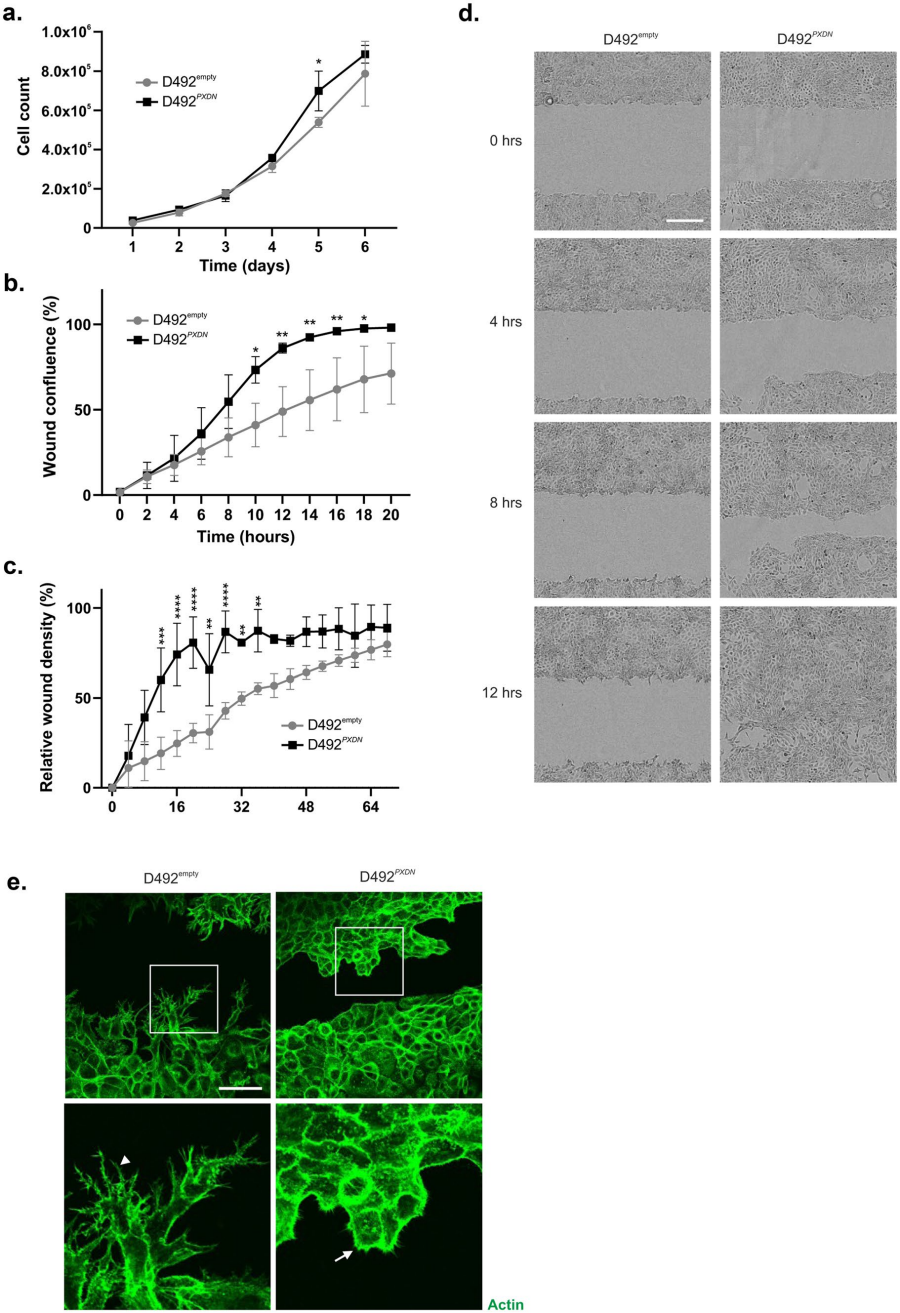
Overexpression of *PXDN* increased proliferation, decreased wound closure time and negatively affected plasticity in D492 cells. **a** *PXDN* did not significantly affect proliferation rate in D492 cells. D492^*PXDN*^ and D492^empty^ cells proliferated at a similar rate. D492^*PXDN*^ only proliferated slightly faster on day 5 of the experiment. Statistical significance was determined by two-way ANOVA with multiple comparisons (*p ≤ 0.05) and data is presented as an average of three replicated experiments (mean ± SD). **b** D492^*PXDN*^ cells had increased migratory capacity in wound healing. *PXDN* significantly increased migration in D492 cells in wound healing compared to D492^empty^. Data was analyzed in IncuCyte Zoom and is presented as wound confluence % and an average of three replicated experiments (mean ± SD). Statistical significance was determined by two-way ANOVA with multiple comparisons (*p ≤ 0.05, **p ≤ 0.01). **c** D492^*PXDN*^ cells had increased invasive capacity in wound healing under Matrigel. D492^*PXDN*^ cells wound closure was significantly accelerated compared to D492^empty^ when Matrigel was applied on top of cells after scratching. Data was analyzed in IncuCyte Zoom and is presented as wound confluence % and an average of three replicated experiments (mean ± SD). Statistical significance was determined by two-way ANOVA with multiple comparisons (**p ≤ 0.01, ***p ≤ 0.001, ****p ≤ 0.0001). **d** D492^*PXDN*^ cells closed the wound in 12 hours in invasion scratch assay under Matrigel. D492^*PXDN*^ cells were able to invade through Matrigel and close scratch wound area in 12 hours. In comparison D492^empty^ cells reached wound closure four and half days later. Scale bar = 300 µm. **e** D492^*PXDN*^ formed lamellipodia during wound closure under Matrigel. Immunofluorescence staining of actin filaments in wound healing under Matrigel, 24 hours post scratching, revealed that D492^empty^ formed extensive filopodia (example depicted with arrowhead) while D492^*PXDN*^ cells formed lamellipodia (example depicted with arrow). Furthermore, peripheral actin staining was increased in D492^*PXDN*^. Scale bar = 150 µm

Next, we applied a series of functional assays to clarify whether changes in phenotype, as a result of *PXDN* overexpression, also affected cell behaviour. There was no significant difference in proliferation in D492^PXDN^ compared to D492^empty^, except at a single timepoint (Fig. 5a). However, *PXDN* promoted migration of D492 cells, evidenced by increased migratory ability of D492^*PXDN*^ in a wound healing assay, where wound confluence reached near 100% 15 hours after application of the scratch wound. On the contrary, when the experiment was terminated at 20 hours, D492^empty^ confluency was still only near 75% (Fig. 5b) Invasion assay revealed a similar trend where wound edges under Matrigel closed significantly sooner in D492^*PXDN*^ compared to D492^empty^ (Fig. 5c, d). Immunofluorescence staining of actin showed that D492^empty^ cells formed long, branching filopodia that were absent in D492^*PXDN*^. However, D492^*PXDN*^ formed lamellipodia with stronger peripheral actin signal than D492^empty^ (Fig. 5e). As phenotype can influence response to chemically induced apoptosis, we performed apoptosis assay using Camptotechin. This revealed significant increase in sensitivity to apoptosis in D492^*PXDN*^ compared to D492^empty^ (Supplementary Fig. 3).

### Basal Markers were Significantly Upregulated in D492^***PXDN***^ cells

**Fig. 6:**
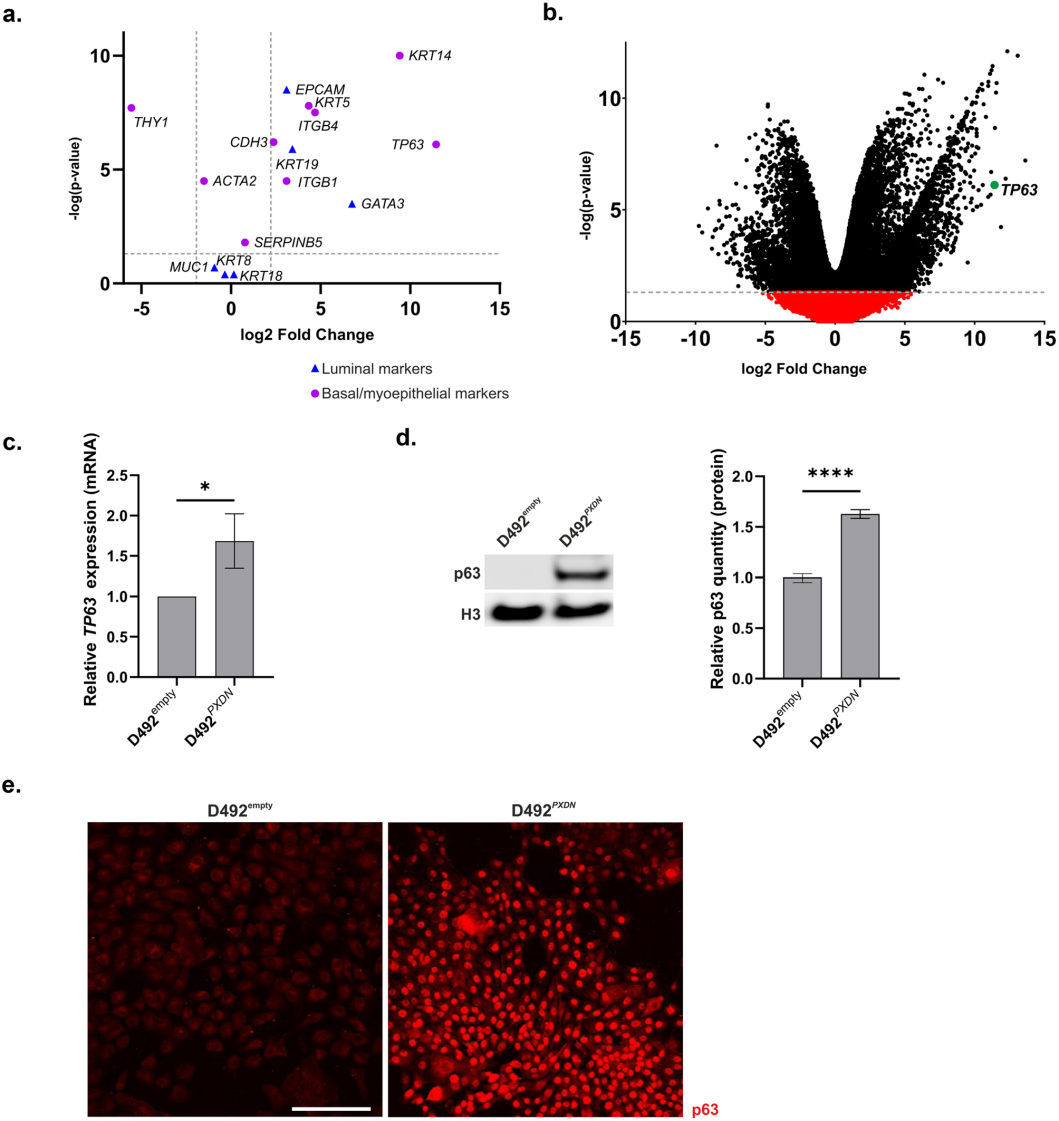
Basal phenotype was induced in D492^*PXDN*^ cells. **a** Basal markers were more significantly upregulated in D492^*PXDN*^ than luminal and myoepithelial markers. Volcano plot depicting differential gene expression of basal, luminal and myoepithelial markers in D492^*PXDN*^ compared to D492^empty^. Dotted lines mark statistical significance of p-value=0.05 and log2 fold change above 2 and below -2. **b** *TP63* was one of the most upregulated genes in D492^*PXDN*^. Volcano plot showing RNA sequencing results from D492^empty^ and D492^*PXDN*^ in monolayer. Significantly upregulated genes (*p*≤ 0.05) are represented by black dots and non-signifcant genes are represented by red dots. **c** PXDN overexpression induced upregulation of p63. qRT-PCR revealed significant upregulation of basal cell marker p63 in D492^*PXDN*^. Statistical significance in qRT-PCR was determined by unpaired Student‘s t-test (*p ≤ 0.05) and data is presented as an average of three replicated experiments (mean ± SD). **d** Protein level upregulation of p63 was confirmed by Western blot. Relative quantification of protein band is displayed to the right. Statistical significance of Western blot was determined by unpaired Student‘s t-test (****p ≤ 0.0001) and data is presented as an average of three replicated experiments (mean ± SD). **e** Immunofluorescence staining of D492^empty^ and D492^*PXDN*^ revealed increased nuclear signal of p63 in D492^*PXDN*^. Upregulation of p63 was confirmed by Western blot of nuclear protein. Scale bar = 125 µm

To gain insight into how overexpression of *PXDN* influences gene expression we performed RNA sequencing on D492^empty^ and D492^*PXDN*^ in monolayer and in 3D culture. Results from monolayer showed that *KRT5* and *KRT14* were among the most significantly upregulated genes in D492^*PXDN*^ compared to D492^empty^. Further analysis of commonly applied basal, luminal and myoepithelial markers in breast epithelium showed that, compared to luminal and myoepithelial markers, the basal markers were more highly expressed with higher level of significance (Fig. 6a). *GATA3*, an important transcription factor involved in luminal epithelial cell differentiation [30], was significantly upregulated in D492^*PXDN*^, although it did not seem to be sufficient in inducing the luminal phenotype (Fig. 6a). Interestingly, the basal marker *TP63* (p63), a known transcription factor for *KRT14* and a key player in epithelial differentiation in the mammary gland [16, 31, 32], was one of the highest upregulated genes in D492^*PXDN*^ (Fig. 6b). This was confirmed on the mRNA- and protein-level with quantitative RT-PCR (Fig. 6c) and Western blot of nuclear fraction protein (Fig. 6d), respectively. Furthermore, immunofluorescence staining showed increased protein level expression and nuclear localization of p63 in all D492^*PXDN*^ cells, indicating increased activity (Fig. 6e). To investigate whether the basal shift in phenotype observed in D492^*PXDN*^ cells was due to *TP63* function we performed transient knock down of *TP63* with siRNA. Successful knock down of *TP63* was confirmed with quantitative RT-PCR and immunofluorescence staining (Supplementary Fig. 4a). Interestingly, the knock down did not significantly affect expression of *PXDN* (Supplementary Fig. 4b) or basal keratins *KRT5/6* and *KRT14* in D492^*PXDN-*KD *TP63*^ compared to D492^*PXDN-*Ctrl^. *KRT19* showed a trend towards upregulation in D492^*PXDN-*KD *TP63*^ although the change was not significant. Immunofluorescence staining did not show any protein level change in keratins between D492^*PXDN-*KD *TP63*^ and D492^*PXDN-*Ctrl^ (Supplementary Fig 4c). Furthermore, transient knock down of *PXDN* in D492 cells did not significantly alter the expression of *KRT14, KRT19* or *TP63* (Supplementary Fig. 5).

### *PXDN* was Involved in Epithelial Differentiation and Suppressed EMT

**Fig. 7:**
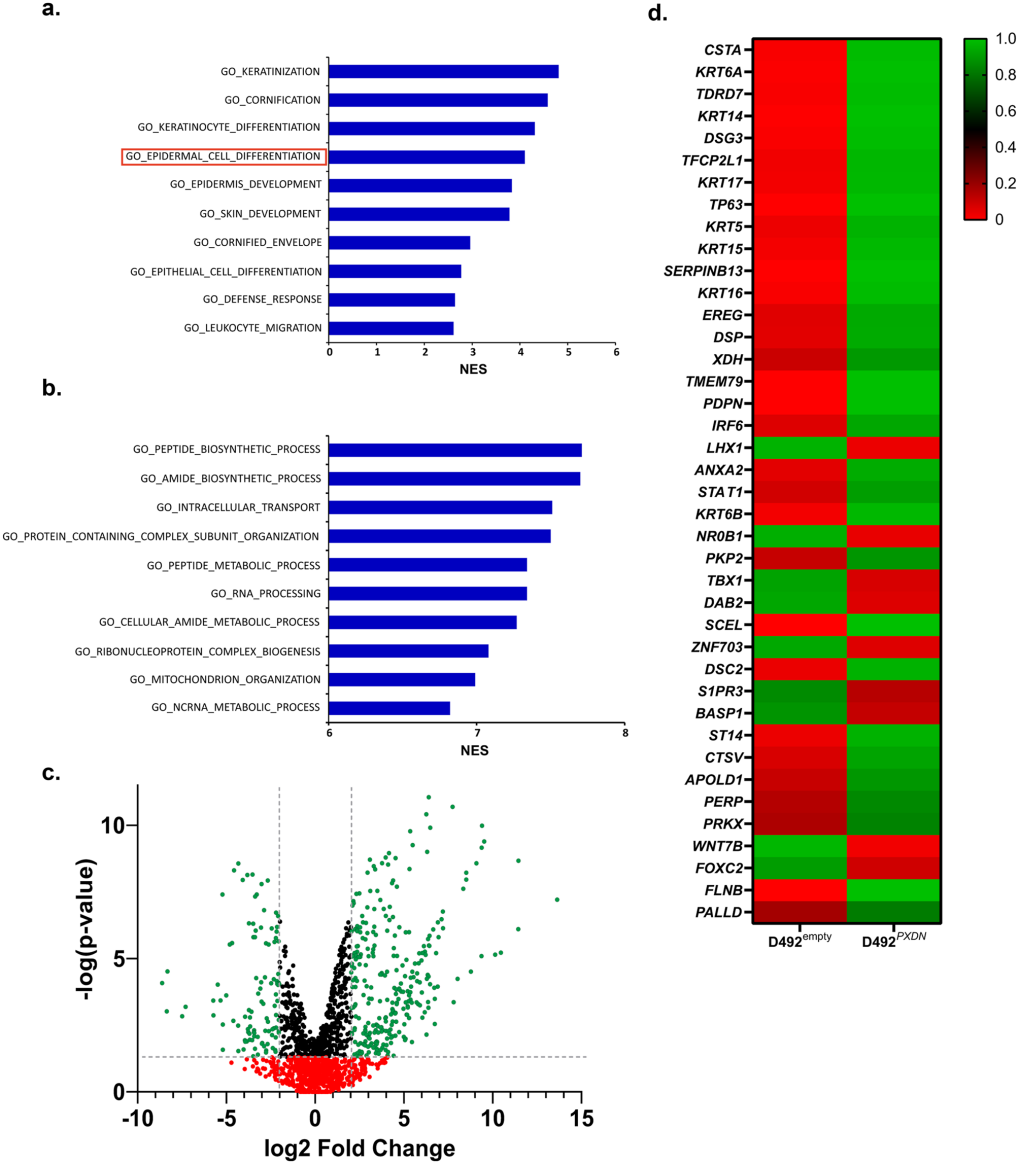
RNA-sequencing revealed enrichment of genes involved in epithelial cell differentiation and development. **a** Developmental and differentiation processes were enriched in D492^*PXDN*^ in 2D. GSEA results using GO: Gene Ontology dataset showed that processes involved in epithelial development and differentiation were significantly enriched (FDR *q*≤0.05), most notably the GO_EPITHELIAL_CELL_DIFFERENTIATION gene set. NES: Normalised Expression Score. **b** Metabolic pathways were most enriched in D492^*PXDN*^ in 3D. GSEA results using GO: Gene Ontology dataset showed that processes involved in metabolism were most significantly enriched (FDR *q*≤0.05). NES: Normalised Expression Score. **c** Approximately two thirds of the genes belonging to the GO_EPITHELIAL_CELL_DIFFERENTIATION gene set, that were significantly differentially expressed, were upregulated in D492^*PXDN*^. Volcano plot representing genes in the GO_EPITHELIAL_CELL_DIFFERENTIATION gene set in D492^*PXDN*^ cells. **d** In the top 40 most significantly differentiated genes belonging to the GO_EPITHELIAL_CELL_DIFFERENTIATION gene set the majority was upregulated in D492^*PXDN*^. Heatmap representing the top 40 most significantly differentially expressed genes in the GO_EPITHELIAL_CELL_DIFFERENTIATION gene set. Among them are *KRT6A, KRT5, KRT14* and *TP63*

Next, as phenotypic shift was already established in D492^*PXDN*^, we were interested in seeing what biological processes were affected by overexpression of *PXDN.* We performed pre-ranked Gene Set Enrichment Analysis (GSEA) using the GO: Gene Ontology datasets which showed that various epithelial developmental processes were significantly enriched, especially those related to cornification, keratinization and development of skin and epidermis (Fig. 7a). As it is generally accepted that the mammary gland is a modified sweat gland that arises from pluripotent epidermal stem cells of the skin [33, 34], common developmental pathways between the two organs are not surprising. More interestingly, GO_EPITHELIAL_CELL_DIFFERENTIATION was significantly enriched. In this category, 113 genes were significantly downregulated (p<0.05, log2FC≤-2.0) while 300 genes were significantly upregulated (p<0.05, log2FC≥2.0), contributing to the enrichment observed in the results (Fig. 7b). Of the top 40 most highly differentially expressed genes belonging to the GO_EPITHELIAL_CELL_DIFFERENTIATION gene set most were upregulated in D492^*PXDN*^ (Fig. 7c).

**Fig. 8:**
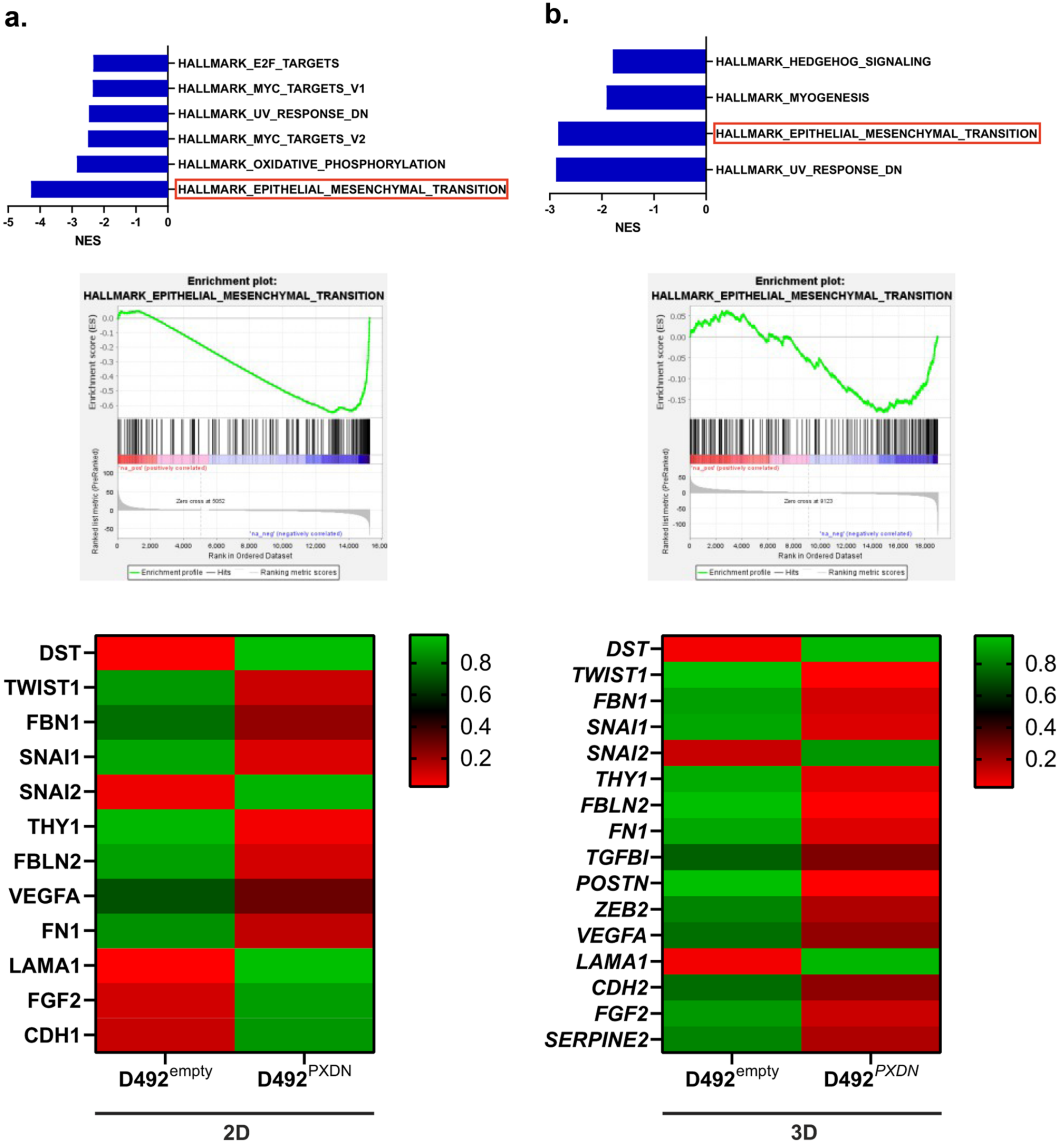
*PXDN* suppressed epithelial to mesenchymal activity and led to significant downregulation of key EMT factors. **a** EMT was significantly negatively enriched and key EMT factors were significantly downregulated in D492^*PXDN*^ in 2D culture. GSEA results using the HALLMARK data set revealed significant negative enrichment of HALLMARK_EPITHELIAL_MESENCHYMAL_TRANSITION gene set (FDR q-value≤0.05). Heatmap depicting key significantly differentially expressed factors involved in EMT in 2D culture. NES: Normalised Expression Score. **b** EMT was significantly negatively enriched and key EMT factors were significantly downregulated in D492^*PXDN*^ 3D culture. HALLMARK_EPITHELIAL_MESENCHYMAL_TRANSITION was the second most negatively enriched gene set (FDR q-value≤0.05) in D492^*PXDN*^ colonies cultured in 3D-rBM. Heatmap depicting key significantly differentially expressed factors involved in EMT in 3D culture. NES: Normalised Expression Score

Next, we performed pre-ranked GSEA analysis using the HALLMARK dataset. Monolayer data showed significant negative enrichment of HALLMARK_EPITHELIAL_TO_MESENCHYMAL_TRANSITION. Furthermore, several EMT factors were significantly downregulated in D492 ^*PXDN*^ (Fig. 8a). As epithelial to mesenchymal transition (EMT) contributes to epithelial plasticity and is a fundamental process in branching morphogenesis [15, 35], we were interested in whether this would also be the case in D492^*PXDN*^ 3D colonies, where branching was inhibited. We therefore performed RNA sequencing on D492^empty^ and D492^*PXDN*^ colonies isolated from 3D-rBM. Results showed that EMT was the second most negatively enriched pathway in D492^*PXDN*^. Furthermore, a greater number of EMT factors was downregulated in 3D compared to 2D conditions. (Fig. 8b). These results indicate that *PXDN* is involved in suppression of EMT which might contribute to inhibition of branching morphogenesis.

### PXDN in Breast Cancer

Immunohistochemistry of three subtypes of breast cancer (ER-positive, HER2-positive and Triple negative) revealed that PXDN was expressed in cancer cells. Furthermore, four tumors showed PXDN-positive fibrils in the tumor stroma (Supplementary Fig. 6a). Results from the Gene expression-based Outcome for Breast Cancer Online (GOBO) database showed that women with basal and HER2-positive tumors that had high *PXDN* expression had poorer distant metastasis free survival (DMFS) compared to women with low *PXDN*-expressing tumors (Supplementary Fig. 6b).

## Discussion

Until now the presence and role of *PXDN* within the human breast has not been explored. In this study we show, for the first time, that *PXDN* is expressed within both the epithelial and stromal compartments of the breast, in LEPs and MEPs as well as fibroblasts and BRENCs, respectively. This was confirmed with immunohistochemistry of paraffin embedded normal breast tissue, where PXDN expression was detected within the epithelium and stroma.

In the past two decades lineage tracing studies have attempted to delineate distinct cell subpopulations within the mammary gland, with the purpose of identifying the hierarchy of stem and progenitor cells responsible for epithelial development and maintenance. Cells belonging to the basal lineage have thus far been recognized as the main instigators of differentiation [8, 10, 36, 37], and the implantation of a single pluripotent stem cell into cleared mouse fat pad can reconstruct a full mammary tree [38]. Villadsen et al. [2] identified the multipotent stem cell populations according to co-expression of KRT14 and KRT19. Earlier on, Yang et al. [39] had reported that p63, a member of the p53 family, was essential for basal cell population maintenance and epithelial development. Furthermore, they showed that p63 deficient mice underwent non-regenerative terminal epithelial differentiation and lacked certain epithelial structures such as the mammary gland. In previous work, members of our group showed that cell plasticity, with the ability to form cells with both luminal and myoepithelial characteristics, was necessary for branching morphogenesis in D492 [16]. Furthermore, they found that along with the mesenchymal repressor miR-200c-141, p63 reverted D492M, a mesenchymal daughter cell line of D492, back to epithelial phenotype. This restored cell plasticity and thus branching morphogenesis in 3D culture. On the contrary overexpression of p63 in primary luminal epithelial cells induced expression of basal markers and basal phenotype [40]. In the present study, we show that overexpression of *PXDN* in the epithelial progenitor cell line D492 induces basal phenotype and leads to loss of plasticity, inhibiting the cell line from flexibly forming cells with luminal and myoepithelial characteristics and thereby inhibiting branching morphogenesis. This is highlighted by co-expression of *KRT14, KRT19* and p63, which reflects the expression pattern of mammary epithelial cell subpopulations that have previously been identified as mammary progenitor cells [41, 42]. Interestingly, transient silencing of *TP63* did not lead to reduced expression of the basal keratins. This indicates that, despite its significant upregulation in D492^*PXDN*^, p63 is not the primary driver of the basal phenotype in these cells. It is also unlikely that p63 knock down could reverse plasticity inhibition in D492^*PXDN*^, thereby restoring branching, as some level of p63 activity is needed for necessary cell differentiation to occur [16]. This is supported by the fact that p63 knock down in VA10, a bronchial epithelial cell line with basal characteristics that forms differentiated bronchial epithelium in air-liquid interface culture, inhibited epithelial differentiation and induced senescence [43]. The role of other mediators of basal phenotype are to be explored in further studies. Another aspect of branching morphogenesis is the activity of FGFR2, which is critical during mammary gland development in mice along with FGFR1 [44–46]. Lack of *FGFR2* expression in basal cells of the murine epithelium leads to failure in luminal epithelial cell differentiation and inhibition of branching morphogenesis [47, 48]. As *FGFR2* was significantly downregulated in D492^*PXDN*^, both in 2D and 3D (Supplementary Fig. 7), this could explain inhibition of both cell plasticity and branching in 3D. However, this remains a subject for future investigations.

While D492^*PXDN*^ cells attain a basal phenotype, they do not show signs of increased EMT as was demonstrated with RNA sequencing. Physiological levels of *PXDN* in D492 cells are very low (Fig. 2a). However, a previous study showed that *PXDN* expression is significantly upregulated in the mesenchymal D492M cell line [17]. In that study, knock down of PXDN in D492M reduced mesenchymal traits in the cells, resulting in a shift towards epithelial phenotype. This might indicate divergent roles of *PXDN* in D492M, where *PXDN* expression seems to contribute to mesenchymal traits, as opposed to D492 cells where overexpression of *PXDN* results in downregulation of EMT factors. Inhibition of EMT might seem contradictive of the decreased wound closure time observed in the cells during live cell imaging, albeit without the formation of cell protrusions often connected to cell migration [49, 50]. However, epithelial cells can achieve increased motility without losing E-cadherin expression and the epithelial phenotype and without the formation of leading cellular protrusions, by undergoing collective migration [11], which is one of key elements involved in branching morphogenesis in mammary gland development [11, 51, 52]. Collective migration and EMT are two distinct mechanisms that can occur independently although between them there is a spectrum of cell behaviour that can result in a combination of the two [52]. Cheung et al. [53] showed that the basal phenotype, with upregulation of *KRT14* and p63, was essential to collective migration without EMT. Furthermore, induced expression of ΔNp63, an isoform of p63, increased migration in breast cancer cells while inhibiting the formation of cell protrusions [54]. ΔNp63 has also been linked to selective regulation of EMT factors in breast cancer cells, as upregulation of *SNAI2* lead to increased migration while miR-205 mediated inhibition of *ZEB1* and *ZEB2* resulted in EMT suppression [55]. Interestingly, RNA-sequencing of D492^*PXDN*^ also revealed upregulation of *SNAI2* and downregulation of *ZEB2* which could contribute to the observed phenotype. Cell death is often included in functional analysis of mammary epithelial cell lines as it is often influenced by change in phenotype [56]. A possible mechanism behind the difference in cell death between D492^PXDN^ and D492^empty^ might be found in the peroxidase activity of PXDN. The peroxidase domain of PXDN utilizes H_2_O_2_ and yields hypochlorous acid, which is a strong oxidant and an inducer of oxidative stress through the generation of reactive oxygen species (ROS) [57–59]. Camptotechin utilizes ROS to provoke cell cycle arrest and apoptosis [60]. Increased expression of PXDN in D492 cells could lead to increased production of ROS which are then readily available to Camptotechin, thereby increasing cell death.

The role of PXDN in breast cancer is previously unexplored. The reason why PXDN only affects DMFS in basal and HER2-positive breast cancers, while also being expressed in estrogen (ER)-positive tumors, is unclear. Triple-negative breast cancers, named by their lack of expression of ER, progesterone and HER2, are typically basal-like and are associated with aggressiveness and poor prognosis [61]. Furthermore, a basal subtype of HER2-positive breast cancers is also related to reduced survival and resistance to therapy [62]. Whether increased expression of *PXDN* could contribute to basal characteristics within these breast cancer subtypes, thereby causing increased metastatic potential, therapy resistance or both, remains a topic for further studies.

Despite being present in both layers of the epithelium *in vivo*, *PXDN* inhibits plasticity in D492 cells and only induces basal phenotype. This cannot be explained effectively with the already known role of *PXDN* as a crosslinker of collagen IV within the extracellular matrix [20], although the basement membrane plays an important regulatory role in epithelial development along with other microenvironmental elements [63, 64]. We therefore propose a novel function of *PXDN* in progenitor cells as an inducer of p63 and basal phenotype and a suppressor of cell plasticity and EMT, providing evidence that *PXDN* might hold a modulatory role in mammary gland development.

## Supplementary Information

Below is the link to the electronic supplementary material.

Supplementary file1 (PDF 529 KB)

Supplementary file2 (DOCX 21 KB)
