# Correction to: Peroxidasin Enhances Basal Phenotype and Inhibits Branching Morphogenesis in Breast Epithelial Progenitor Cell Line D492

**DOI:** 10.1007/s10911-022-09513-x

**Published:** 2022-02-12

**Authors:** Anna Karen Sigurdardottir, Arna Steinunn Jonasdottir, Arni Asbjarnarson, Hildur Run Helgudottir, Thorarinn Gudjonsson, Gunnhildur Asta Traustadottir

**Affiliations:** 1 Stem Cell Research Unit, Biomedical Center, Department of Anatomy, Faculty of Medicine, School of Health Sciences, University of Iceland, Reykjavik, Iceland; 2 Department of Laboratory Haematology, Landspitali – University Hospital, Reykjavik, Iceland

## Correction to: Journal of Mammary Gland Biology and Neoplasia https://doi.org/10.1007/s10911-021-09507-1

Due to an error during typesetting, an incomplete Supplementary file was originally published with this article. Supplementary figures were missing and this has now been uploaded.

The original article has been corrected.

