# Supplementary material for: Peroxidasin Enhances Basal Phenotype and Inhibits Branching Morphogenesis in Breast Epithelial Progenitor Cell Line D492
Source: J Mammary Gland Biol Neoplasia. 2021 Dec 28;26(4):321–38. doi: 10.1007/s10911-021-09507-1 (PMC8858314; doi:10.1007/s10911-021-09507-1)

Supplementary fig. 1

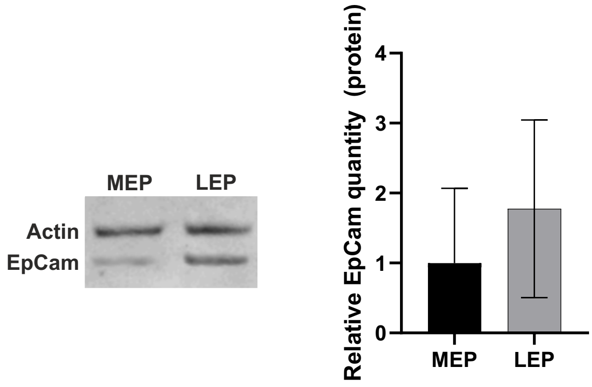

Supplementary fig. 2

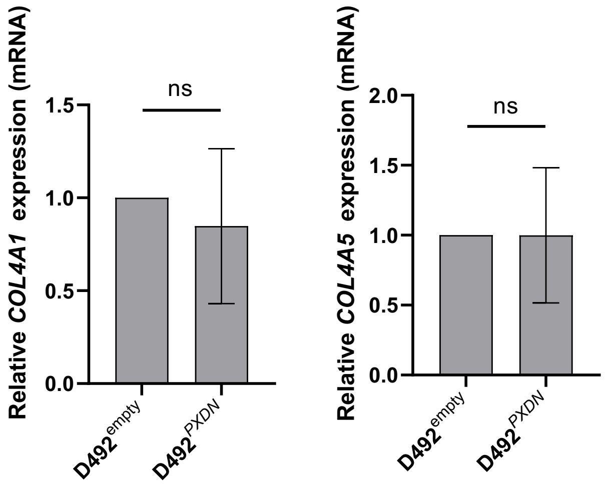

Supplementary fig. 3

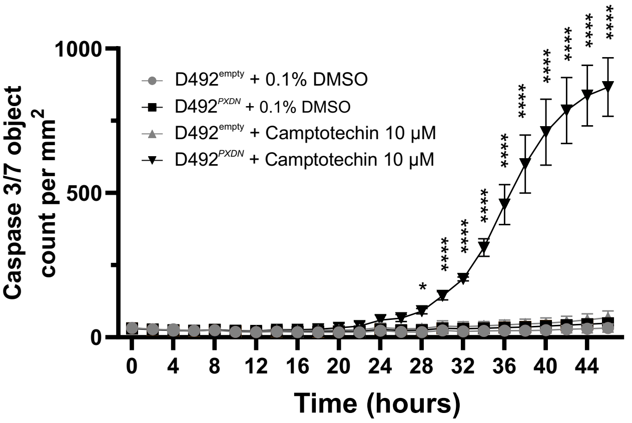

Supplementary fig. 4

a.

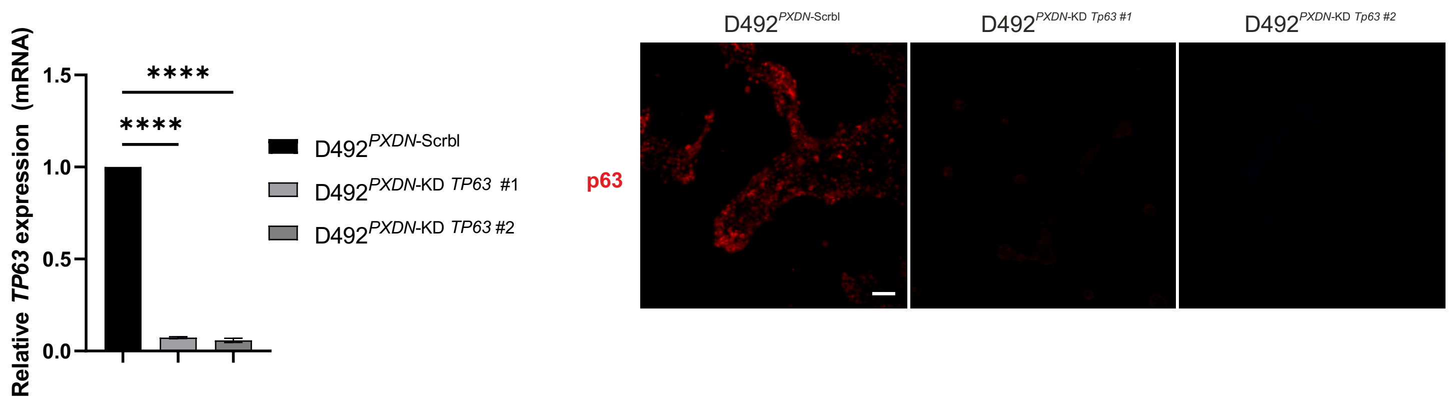

b.

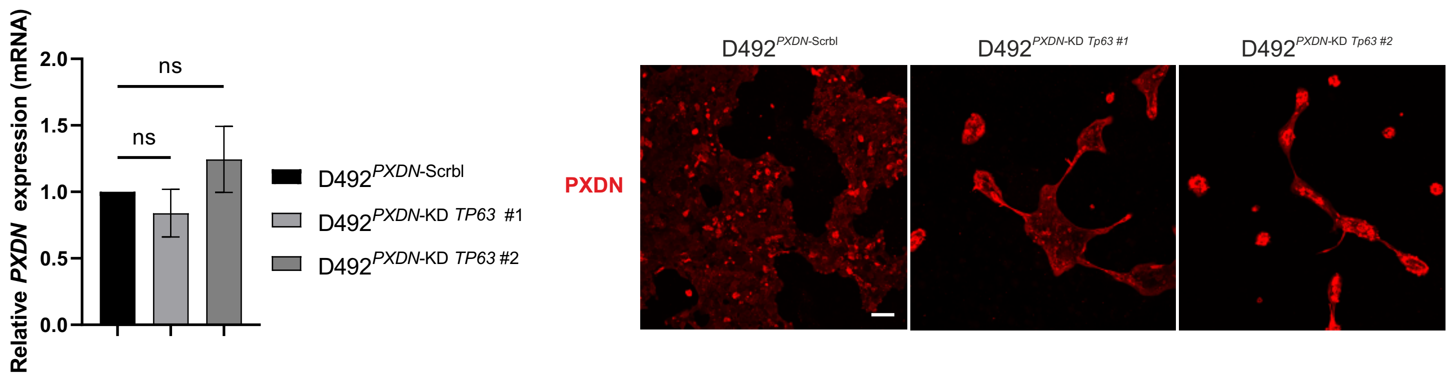

c.

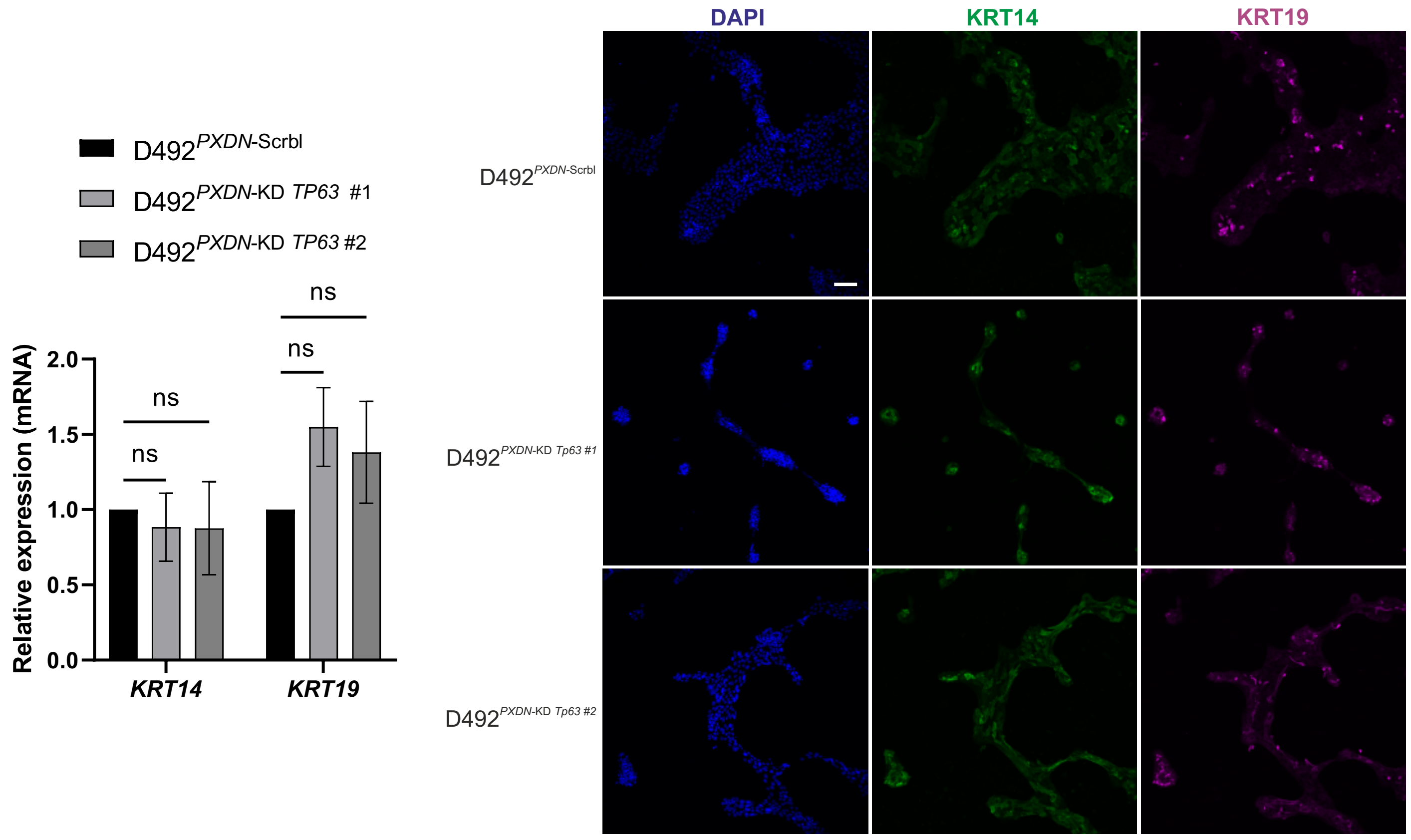

Supplementary fig. 5

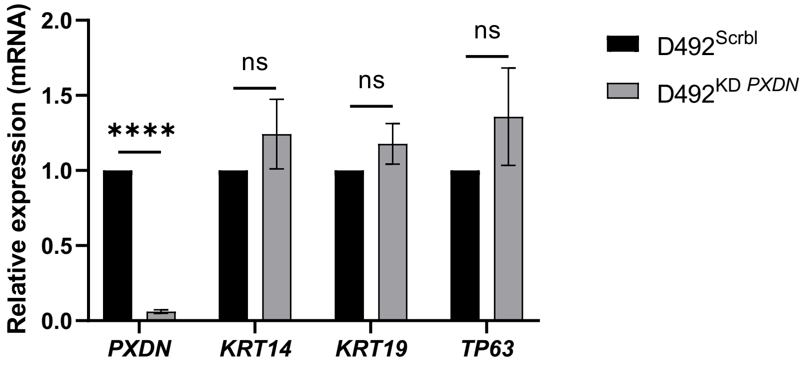

Supplementary fig. 6

a.

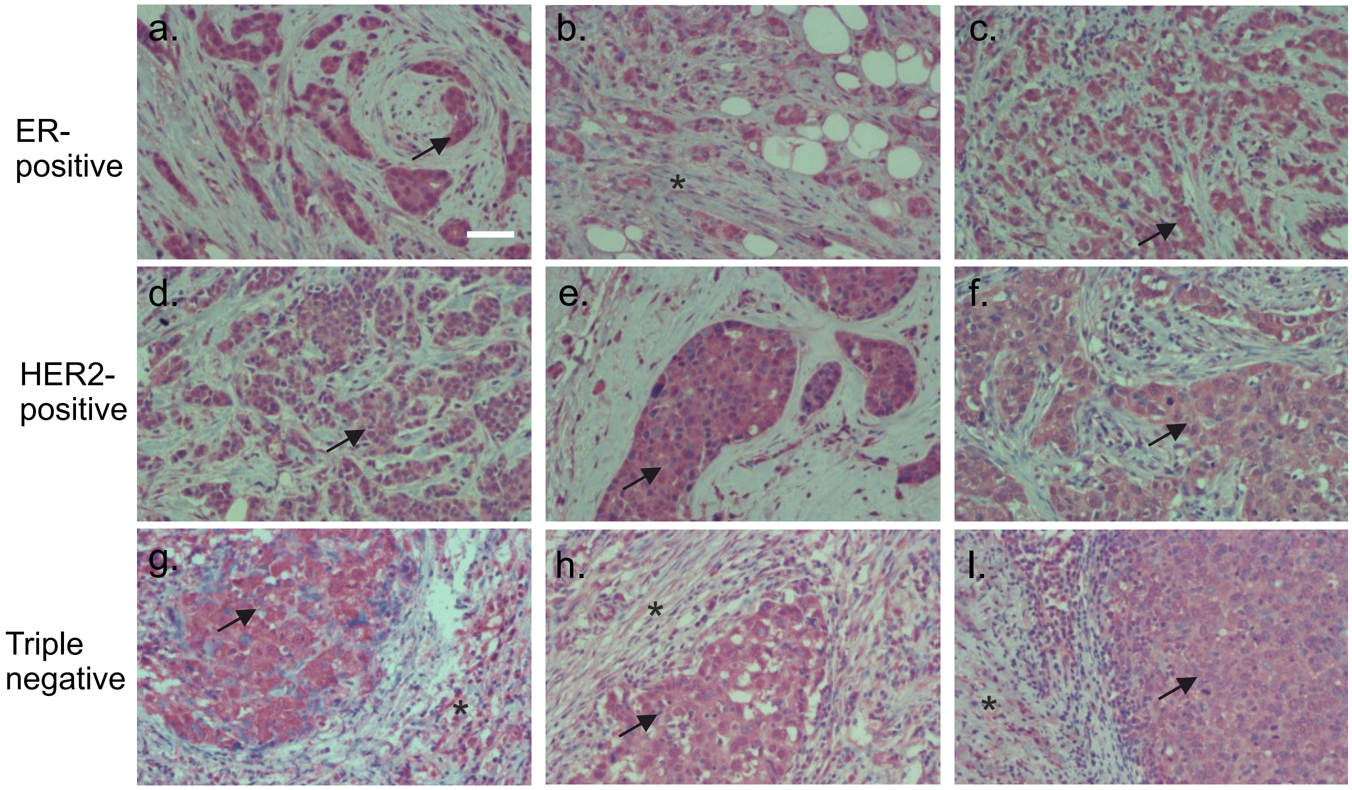

b.

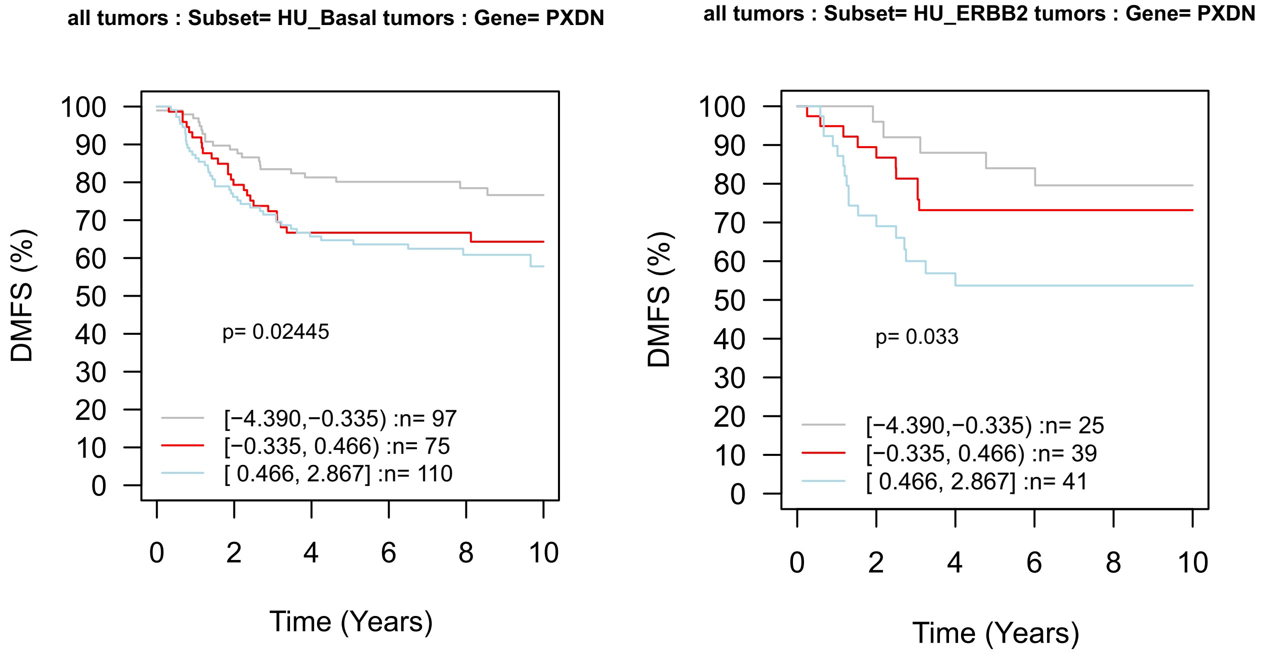

Supplementary fig. 7

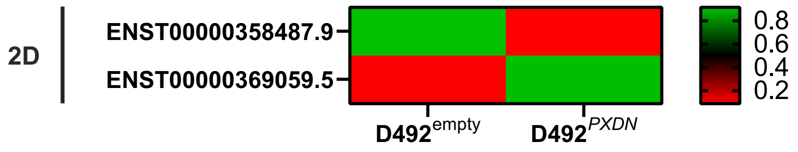

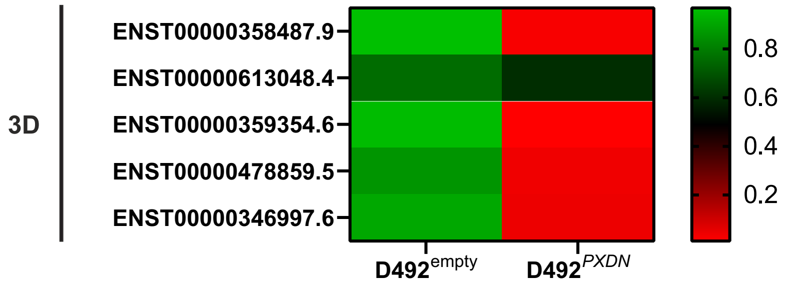

Supplement: Supplementary file 1 — Supplementary file1 (PDF 529 KB) [file 10911_2021_9507_MOESM1_ESM.pdf]
