# Supplementary material for: Peroxidasin Enhances Basal Phenotype and Inhibits Branching Morphogenesis in Breast Epithelial Progenitor Cell Line D492
Source: J Mammary Gland Biol Neoplasia. 2021 Dec 28;26(4):321–38. doi: 10.1007/s10911-021-09507-1 (PMC8858314; doi:10.1007/s10911-021-09507-1)
Supplement: Supplementary file 2 — Supplementary file2 (DOCX 21 KB) [file 10911_2021_9507_MOESM2_ESM.docx]

**Supplementary fig. 1** Western blot confirming magnetic sorting of EpCam-positive (LEP) and EpCam-negative (MEP) primary mammary epithelial cells.

**Supplementary fig. 2** Overexpression of *PXDN* did not signficantly alter expression of collagen IV genes. Expression of *COL4A1* and *COL4A5* did not change significantly in D492*^PXDN^* compared to D492^empty^. Statistical significance in qRT-PCR was determined by unpaired Student‘s t-test and data is presented as an average of three replicated experiments (mean ± SD).

**Supplementary fig. 3** D492*^PXDN^* cells were more sensitive to chemically induced apoptosis. *PXDN* increased sensitivity to chemically induced apoptosis in D492 cells when treated with Camptotechin. Data was analyzed in IncuCyte Zoom and is presented as Caspase 3/7 object count per mm^2^. Statistical significance was determined using two-way ANOVA followed by Tukey‘s post hoc analysis (**p* ≤ 0.05, *****p* ≤ 0.0001) and data is presented as an average of three independent experiments (mean ± SD, n=3).

**Supplementary fig. 4** Transient knock down of *TP63* in D492*^PXDN^* did not significantly affect expression of *PXDN* or keratins

1. Confirmation of transient knock down of *TP63* in D492*^PXDN^* with two different siRNAs with qRT-PCR and immunofluoresence staining. Statistical significance was determined using One-way ANOVA (*****p* ≤ 0.0001) and data is presented as an average of three replicated experiments (mean ± SD). Scale bar = 100 µm.
2. *TP63* knock down did not significantly alter expression of *PXDN* as measured via qRT-PCR and immunofluoresence staining did not show a difference in PXDN signal intensity. Statistical significance was determined using One-way ANOVA and data is presented as an average of three replicated experiments (mean ± SD). Scale bar = 100 µm.
3. *TP63* knock down did not significantly affect expression of *KRT14 or KRT19* as measured via qRT-PCR and immunofluoresence staining did not show a difference in signal intensity. Statistical significance was determined using One-way ANOVA and data is presented as an average of three replicated experiments (mean ± SD). Scale bar = 100 µm.

**Supplementary fig. 5** Transient siRNA knock down of *PXDN* in D492 did not significantly affect expression of keratins or *TP63*. qRT-PCR confirmed siRNA mediated transient knock down of *PXDN* in D492 cells and also revealed no significant change in expression of *KRT14, KRT19*  and *TP63.* Statistical significance in qRT-PCR was determined by unpaired Student‘s t-test (*****p* ≤ 0.0001) and data is presented as an average of three replicated experiments (mean ± SD).

**Supplementary fig. 6** *PXDN* expression in breast cancer

1. PXDN was expressed in three different breast cancer subtypes: ER-positive, HER2-positive and Triple negative. Immunohistochemistry on paraffin embedded tumor tissue revealed positive PXDN signal (red) in all cancer cells (examples depicted with arrows) in all tumors. Tumors b., g., h. and i. also had PXDN-positive fibrils in the intratumor stroma (asterix). Tissue slides were counterstained with hematoxylin. Scale bar = 100 µm.
2. Women with basal tumors and HER2-positive breast cancers that express high levels of *PXDN* had significantly lower distant metastatsis free survival (DMFS) than women with low *PXDN* expressing tumors. Data from the GOBO database.

**Supplementary fig. 7** Heatmaps showing differential expression of *FGFR2* transcripts in D492^empty^ and D492*^PXDN^* in 2D and 3D. In 2D, one of two significantly differentially expressed transcripts was downregulated in D492*^PXDN^*. However, all transcripts were downregulated in D492*^PXDN^* in 3D.
